# Low-Income Asian Americans: High Levels Of Food Insecurity And Low Participation In The CalFresh Nutrition Program

**DOI:** 10.1377/hlthaff.2023.00116

**Published:** 2023-09-20

**Authors:** Milkie Vu, Duy Trinh, Namratha R. Kandula, Nhat-Ha Tran Pham, Jennifer Makelarski, Hilary K. Seligman

**Affiliations:** Northwestern University, Chicago, Illinois.; Princeton University, Princeton, New Jersey.; Northwestern University.; University of Pennsylvania, Philadelphia, Pennsylvania.; independent researcher, Chicago, Illinois.; University of California San Francisco, San Francisco, California.

## Abstract

Little is known about food insecurity and the extent of Supplemental Nutrition Assistance Program (SNAP) participation in the heterogeneous Asian American population. Using California Health Interview Survey data from the period 2011–20, we examined both issues among low-income Asian American adults from six origin groups: Chinese, Filipino, Japanese, Korean, South Asian, and Vietnamese. We found high and varied levels of overall food insecurity, with the highest burden among Filipino adults (40 percent). Food insecurity by severity was also heterogenous; very low food security affected 2 percent of Chinese adults but 9 percent and 10 percent of Filipino and Japanese adults, respectively. Participation in CalFresh (California-implemented SNAP) ranged from 11 percent and 12 percent among Korean and Chinese adults, respectively, to 20 percent among Vietnamese adults. Compared with English-proficient low-income Asian American adults, those with limited English proficiency were no less likely to participate in CalFresh, possibly reflecting language assistance required by California law and provided by community-based organizations. These results underscore the importance of collecting and reporting disaggregated data by Asian origin group that could inform targeted outreach and interventions.

Food security, or consistent, dependable access to enough food for an active, healthy life,^1^ is a critical determinant of health.^2–4^ Participation in US nutrition assistance programs (for example, the Supplemental Nutrition Assistance Program, or SNAP) alleviates food insecurity.^5–7^ However, many eligible individuals and households are not enrolled: SNAP participation among eligible populations was estimated to be 78 percent nationwide during the period October 2019–February 2020.^8^ In the US, there are racial and ethnic disparities in food insecurity; for example, non-Hispanic Black and Hispanic or Latino households experience higher food insecurity (19.8 percent and 16.2 percent, respectively) compared with non-Hispanic White households (7.0 percent).^1^ Emerging research has documented the experiences of nutrition assistance programs for food insecurity among Black, Hispanic or Latino, and American Indian and Alaska Native populations.^9,10^ However, much less is known about these experiences among Asian Americans.

The evidence gaps are partly due to the “model minority” stereotype, which portrays Asian Americans as a homogenous group experiencing high socioeconomic success and little structural racism or disadvantages^11–13^ and, as a consequence, no or little food insecurity. The COVID-19 pandemic shed light on the inaccuracy of such assumptions,^14^ given the thousands of reports in the US of anti-Asian stigma and violence^15^ and recent studies suggesting that Asian Americans experience food acquisition difficulties because of their fear of being racially targeted.^16,17^ Common approaches to data categorization have hampered the ability to explore food insecurity and program enrollment among Asian Americans.^11,14,18^ The limited literature presents aggregated statistics for Asian Americans instead of providing data by origin group (for example, Vietnamese, Chinese, and Korean).^19,20^ However, Asian Americans are a heterogeneous population with diverse origins, languages, socioeconomic status, religious and cultural practices, immigration histories, and patterns of service use.^21–24^ Importantly, different Asian American groups have been found to have varying levels of English proficiency.^25,26^ English proficiency has been linked to both food insecurity and SNAP enrollment in immigrant and refugee populations.^27–32^ Those with limited English proficiency may face challenges in understanding eligibility and recertification requirements and navigating SNAP application processes. For all of these reasons, the use of aggregated data across all Asian origin groups to assess food insecurity and SNAP participation may mask important between-group differences.^11,33^

Data disaggregation can identify which Asian origin group or groups face the highest burden of food insecurity or challenges in SNAP participation. Most population-based surveys on these topics (for example, the National Health and Nutrition Examination Survey) use only English or Spanish questionnaires and do not sample enough Asian Americans from different groups to enable meaningful analyses. However, the California Health Interview Survey (CHIS) is an exception. It surveys people in the state that has the largest population of Asian Americans: Around one-third of Asian Americans live in California.^34,35^ CHIS is also conducted in multiple Asian languages and reaches Asian Americans with limited English proficiency.^36^ Moreover, it reaches samples of Asian Americans from different origin groups that are large enough for analysis.^36^

To our knowledge, only one previous study has used CHIS to explore food insecurity by Asian origin group. That study found that Vietnamese adults experienced higher food insecurity (16 percent) compared with adults in other Asian American origin groups (less than 9 percent for Chinese, Filipino, Japanese, Korean, and South Asian populations).^33^ However, it used data from ten to twenty years ago, and it did not present either disaggregated SNAP participation rates or comparisons with other racial and ethnic groups (for example, Hispanic or Latino), thus limiting its timeliness and potential to inform current policy.

Using CHIS data from the period 2011–20, we examined the prevalence of food insecurity and participation in CalFresh, the California-implemented SNAP that is state supervised and county operated. We focused on low-income Asian Americans from six origin groups: Chinese, Filipino, Japanese, Korean, South Asian, and Vietnamese. These groups are the largest Asian American groups according to census data.^25^ Furthermore, we explored the associations between limited English proficiency and CalFresh participation and assessed whether such associations varied by origin group. The insights gained can help inform—and potentially enhance—program outreach and intervention efforts.

## Study Data And Methods

We used a serial cross-sectional design that pooled data from five consecutive waves of the adult CHIS conducted during 2011–20.

### SAMPLE AND DATA

CHIS is a population-based survey of California’s residential, noninstitutionalized population.^36^ It is the nation’s largest state-level health survey. Each iteration or wave covers two years and collects data from a total of approximately 40,000 adult respondents. Data are collected continuously, with approximately half of the interviews conducted in one calendar year and half in the next calendar year.^36^ The survey has been conducted in English, Spanish, Chinese, Korean, and Vietnamese since 2011 and in Tagalog since 2013.^36^ To our knowledge, no law requires assistance for CHIS interviews in languages other than those listed above. To ensure the representativeness of its data for racial and ethnic groups (including major Asian origin groups), CHIS employs an address-based sample design and divides counties into geographic sampling strata and substrata for probability sampling. Population weights are applied to produce accurate population estimates and totals.^36^

### FOOD INSECURITY AND CALFRESH PARTICIPATION

For respondents with household incomes less than 200 percent of the federal poverty level, CHIS administers the Department of Agriculture’s (USDA’s) six-item Household Food Security Survey Module to assess food security levels.^37^ Those with zero to one affirmative responses to the six questions are categorized as food secure, whereas those with two or more affirmative responses are categorized as food insecure, with either low food security (two to four affirmative responses) or very low food security (five to six affirmative responses).^37^

CHIS measures CalFresh participation using the question, “Are you receiving Food Stamp benefits, also known as CalFresh?” Most CalFresh-receiving households are subject to a gross household income determination test, with the maximum gross household income allowed being 200 percent of poverty.^38^ From 2011 to 2018 CHIS assessed CalFresh participation among only those respondents indicating a household income of 300 percent of poverty or less. During the 2019–20 cycle, the CHIS threshold for assessing CalFresh participation was lowered to a household income of 200 percent of poverty or less. To ensure comparability across CHIS cycles and with the CalFresh eligibility requirement, we restricted CalFresh-related analyses to respondents with a household income of 200 percent of poverty or less for the entire study period. To align with the design of CHIS, respondents with a household income exactly at 200 percent of poverty (*n* = 585 unweighted, 73,701 weighted; 0.25 percent of study sample) were included in our analysis of CalFresh participation but not our analysis of food insecurity.

### RACE AND ETHNICITY

Respondents were asked whether they described themselves as one or more of the following races: American Indian or Alaska Native, Asian, Black or African American, Native Hawaiian, other Pacific Islander, or White. They were also asked whether they identified as Hispanic or Latino. Respondents self-identifying as Asian were further asked to indicate their specific origin group or groups, with a list of seventeen possible choices and an option to specify other groups not on the list.

For our analysis, we categorized non-Hispanic or Latino Asian respondents from the six largest origin groups as Chinese, Filipino, Japanese, Korean, South Asian (defined by CHIS as being Bangladeshi, Indian, Nepalese, Pakistani, or Sri Lankan), or Vietnamese. Other racial and ethnic groups of CHIS respondents included in our analyses, for purposes of comparison, were African American, Hispanic or Latino, and White. (In this article, all racial groups are non-Hispanic unless otherwise specified.) Those identifying as Asian but who did not belong to one of the largest six origin groups, or who chose more than one Asian origin group, were categorized as “other Asian or multiple Asian origin groups.”

### ENGLISH PROFICIENCY

CHIS asked respondents, “What languages do you speak at home?” Those who chose languages in addition to or other than English were asked, “Would you say you speak English…?” and given response options of “very well,” “well,” “not well,” or “not at all.” Based on responses to these questions, we categorized respondents as indicating that they either had limited English proficiency (that is, spoke English not well or not at all) or were English proficient (that is, spoke only English or spoke English very well or well).

### SOCIODEMOGRAPHIC COVARIATES

Covariates measured by CHIS and included in our analysis were age, sex, education, employment status, family type, family size, household income (as percent of poverty), and citizenship status. All of these covariates have been linked to SNAP participation in previous research.^39–42^

### STATISTICAL ANALYSES

We pooled 2011–20 data using a CHIS-provided SAS macro. We conducted all subsequent analyses in R. We conducted descriptive analyses to understand unadjusted rates of food insecurity and CalFresh participation by race and ethnicity. We used two-sample *t*-tests to compare unadjusted rates among racial and ethnic groups. We conducted bivariate logistic regressions to explore the relationships between CalFresh participation and race and ethnicity, English proficiency, and other covariates. To investigate whether limited English proficiency hinders CalFresh participation and whether this issue differentially affects Asian origin groups, we conducted multivariate logistic regressions with CalFresh participation as the outcome and included race and ethnicity, limited English proficiency, and the interaction term between the two (controlling for sociodemographic covariates). All analyses used replicate weights provided by CHIS.^43^ We calculated predicted probabilities of CalFresh participation by English proficiency and Asian origin groups from regression results.^44^ Because of COVID-19, the CalFresh program issued certain waivers for eligibility verification and benefits recertification interviews^45^ and increased regular CalFresh benefit amounts and expanded eligibility criteria for students.^46^ As these changes could have affected our results, we conducted a sensitivity analysis restricting our data to the survey cycles collected before the pandemic (2011–18). The cutoff for statistical significance was set at *p* < 0.05.

### LIMITATIONS

We acknowledge several limitations. First, self-reported data were used for CalFresh participation, which can be subject to multiple biases. Second, the generalizability of our findings may be limited, given multiple CHIS data constraints. CHIS assessed food security status among only those with household incomes less than 200 percent of poverty, even though a third of US food-insecure households have incomes above 200 percent of poverty.^47^ CHIS included only California respondents, which may limit generalizability to other states or settings. Third, CHIS does not allow for disaggregation of the category “South Asian.” It is not available in Japanese or any South Asian languages, limiting the participation of those who could participate in surveys in these languages but not English. Finally, CHIS data are cross-sectional; therefore, the statistically significant associations we report do not necessarily imply cause-effect relationships.

## Study Results

Race and ethnicity, English proficiency, and other sociodemographic characteristics of respondents with household incomes of 200 percent of poverty or less, overall and by CalFresh participation, are described in exhibit 1 and online appendix A.^48^ This information is further described for Asian origin groups and other racial and ethnic groups in appendix B.^48^ Numbers of respondents in the full analytical sample, in each two-year CHIS cycle, and in each racial and ethnic group (unweighted and weighted) are in appendixes C and D.^48^

### FOOD INSECURITY

Among Asian American respondents with household incomes less than 200 percent of poverty, food insecurity rates were 26.7 percent for Chinese respondents, 39.5 percent for Filipino respondents, 26.5 percent for Japanese respondents, 25.0 percent for Korean respondents, 26.5 percent for South Asian respondents, and 28.9 percent for Vietnamese respondents (exhibit 2). Except for Filipino respondents, all Asian origin groups had a significantly lower prevalence of food insecurity compared with Hispanic or Latino (43.6 percent) and White (38.7 percent) respondents. All Asian origin groups had a significantly lower prevalence of food insecurity when compared with African American or Black (49.1 percent) respondents. The severity of food insecurity across Asian origin groups was heterogenous; for example, one in fifty Chinese respondents experienced very low food security, compared with one in sixteen Vietnamese, one in eleven Filipino, and one in ten Japanese respondents.

### CALFRESH PARTICIPATION

Among Asian American respondents with household income of 200 percent of poverty or less, CalFresh participation rates were 12.2 percent among Chinese respondents, 14.9 percent among Filipino respondents, 16.0 percent among Japanese respondents, 11.2 percent among Korean respondents, 14.0 percent among South Asian respondents, and 19.5 percent among Vietnamese respondents (exhibit 3). All Asian origin groups had significantly lower CalFresh participation compared with that of African American or Black respondents (29.2 percent). Chinese, Korean, and South Asian respondents had significantly lower CalFresh participation compared with Hispanic or Latino (20.5 percent) and White (18.8 percent) respondents. Even among the subsample of low-income respondents who indicated food insecurity, CalFresh participation among most Asian origin groups was still lower than that indicated by African American or Black, Hispanic or Latino, and White respondents (appendix E).^48^

### PARTICIPATION BY ENGLISH PROFICIENCY AND RACE AND ETHNICITY

In unadjusted descriptive analyses involving Chinese, Filipino, Korean, South Asian, and Vietnamese respondents, respondents with limited English proficiency had higher CalFresh participation rates than English-proficient respondents. The magnitude of those differences varied considerably across Asian origin groups. For example, Chinese respondents with limited English proficiency participated at more than twice the rate of English-proficient Chinese respondents (15.9 percent versus 7.6 percent, respectively), whereas the difference among Vietnamese respondents with limited English proficiency and those who were proficient in English was much smaller (20.3 percent versus 18.0 percent, respectively). This pattern of higher CalFresh participation among respondents with limited English proficiency, however, did not hold for Hispanic and Latino respondents (appendix F).^48^

Multivariable regressions controlling for other sociodemographic variables (model 1, appendix G)^48^ showed that Chinese respondents had significantly lower odds of CalFresh participation compared with Hispanic or Latino respondents (adjusted odds ratio: 0.65; 95% confidence interval: 0.48, 0.89). No significant difference was found for other Asian origin groups. We also did not find a significant relationship between English proficiency and CalFresh participation overall.

To explore whether the relationship between English proficiency and CalFresh participation was stronger for certain Asian origin groups than others, we further interacted English proficiency with race and ethnicity (model 2, appendix G).^48^ In multivariable models stratified by English proficiency, among English-proficient respondents only, Chinese (aOR: 0.36; 95% CI: 0.25, 0.52) and Korean (aOR: 0.36; 95% CI: 0.19, 0.69) respondents had significantly lower odds of CalFresh participation compared with Hispanic or Latino respondents (exhibit 4 and appendix H).^48^ In models stratified by race and ethnicity, English-proficient respondents from all Asian origin groups had lower odds of CalFresh participation compared to respondents with limited English proficiency from the same origin group, but these differences did not reach statistical significance (appendix I).^48^

Differences in the predicted probabilities of CalFresh participation (appendix J)^48^ further illustrated the differences in CalFresh participation by Asian origin group and English proficiency. Among English-proficient respondents, Korean respondents had the lowest (8.6 percent) and Vietnamese respondents had the highest (20.2 percent) probability of CalFresh participation. However, among respondents with limited English proficiency, Chinese respondents had the lowest (20.7 percent) and Filipino respondents had the highest (28.3 percent) probability of CalFresh participation.

In our sensitivity analysis, restricting our data to the survey cycles collected before the COVID-19 pandemic (2011–18) did not substantively change the results (appendixes K–T).^48^ Detailed interpretations and comparisons of the two data analyses (2011–20 versus 2011–18) are in the notes section of each appendix.^48^

## Discussion

Using ten years of representative data from 2011 to 2020, we report disaggregated prevalence of food insecurity among low-income CHIS respondents from six Asian origin groups. At least one in four low-income Asian American adults experienced food insecurity during the study period. Low-income Filipino adults faced the highest burden of food insecurity, at 40 percent. Furthermore, we observed heterogeneity in the severity of food insecurity across Asian origin groups. Very low food security affected 2 percent of low-income Chinese adults but 9 percent of low-income Filipino and 10 percent of low-income Japanese adults. These results fill important data gaps that have contributed to a lack of public awareness of Asian Americans’ experiences with food insecurity.^14^ For example, the annual USDA national food insecurity report, last published in 2022, included separate estimates for White, Black, and Hispanic populations but failed to include either aggregated or disaggregated Asian populations.^1^

Low-income Filipino adults were disproportionately affected by food insecurity in the study period. In addition, although Japanese adults appeared to have a total prevalence of food insecurity similar to that of several other Asian origin groups, Japanese adults had the highest level of very low food security. Although the literature exploring the experience of food insecurity among Japanese adults is very limited, these results conflict with a previous study that found that Vietnamese adults had the highest prevalence of food insecurity among Asian American adults in California.^33^ At the same time, our findings indicate that Japanese adults had the second-highest CalFresh participation rate among the six low-income Asian origin groups. The particularly high prevalence of both very low food security and CalFresh participation among Japanese respondents prompts questions about whether CalFresh participation effectively addresses food insecurity in Japanese communities, thereby highlighting the need for research that examines the food security experiences of Japanese SNAP participants and uses survey instruments in Japanese. This would facilitate a higher representation of Japanese adults with limited English proficiency and provide a deeper understanding of the dynamic relationship between food security and CalFresh participation in this group.

Our finding of high food insecurity among Filipino respondents is supported by the results of two prior studies. One study, using health plan data and published in 2020, found higher food insecurity among Filipino middle-age and older adults when compared with Chinese people.^49^ The second, using data from the National Latino and Asian American Study and published in 2021, showed that Filipino adults had higher food insecurity (41 percent) compared with Vietnamese (26 percent) and Chinese (27 percent) adults.^50^ Future research should investigate multilevel factors underlying the high food insecurity burden among Filipino communities, attending to their unique experiences and histories with colonialism, structural racism, and immigration.^51^

CalFresh participation was low among most Asian origin groups during the study period, with the lowest rates observed in Chinese and Korean communities. Additional studies should explore approaches to reducing barriers to CalFresh enrollment among Asian American adults, particularly in Chinese and Korean communities. Barriers may include cultural stigma, lack of knowledge, complicated application and verification processes, insufficient outreach, and federal immigration policies (for example, fear of the “public charge” rule).^29,52,53^

Contrary to our hypothesis, limited English proficiency was not a barrier to CalFresh participation for Asian American respondents. In fact, in unadjusted analyses, Asian American respondents with limited English proficiency had generally higher participation rates compared with English-proficient Asian American respondents. That the magnitude of difference varied across origin groups underscores the importance of disaggregating data by Asian origin group.

Previous research has linked limited English proficiency in various populations to higher food insecurity,^28,29,31,32^ but very limited research has examined the relationship between English proficiency and SNAP participation among Asian Americans. Our findings suggest that limited English proficiency is not a barrier to CalFresh enrollment among Asian American respondents. This result may be due to California’s language access laws, which require interpreters and written translations of CalFresh program materials in many Asian languages, including Cambodian, Chinese, Farsi, Hindi, Hmong, Japanese, Korean, Lao, Mien, Punjabi, Tagalog, Thai, and Vietnamese. In addition, this finding may also be explained by the role of community-based organizations. These organizations have extensive reach among Asian Americans with limited English proficiency and often offer CalFresh enrollment navigation assistance.^29,54–56^ Asian American adults with limited English proficiency may use community-based organizations’ services more often than their English-proficient counterparts and therefore be more likely to enroll in CalFresh. Further research, preferably longitudinal, is warranted to better understand the impacts of language accessibility policies and community-based organizations’ services on SNAP participation among Asian Americans with limited English proficiency—not only in California but also in other jurisdictions.

Our study responds to a recent joint national effort from the National Institutes of Health, Centers for Disease Control and Prevention (CDC), and USDA, which calls for data disaggregation to identify populations most affected by nutrition insecurity or underuse of assistance programs.^57^ We found that food insecurity and CalFresh participation rates varied among different Asian American communities, with low-income Filipino adults being disproportionately affected by food insecurity and low-income Chinese and Korean adults having the lowest rates of CalFresh participation. Policy makers and practitioners interested in addressing disparities among Asian origin groups or groups within other major, heterogenous populations may consider data disaggregation as a first step toward identifying and addressing unique needs and barriers faced by specific subpopulations. State agencies are ideally positioned to collect and report disaggregated data by Asian origin group for nutrition assistance program participants. One-size-fits-all food insecurity and nutrition program enrollment interventions may have limited utility,^29^ given the diversity of language and culture, socioeconomic status, immigration histories, and acculturation. Instead, culturally tailored, linguistically appropriate interventions that incorporate targeted outreach, consider unique needs and assets, and involve partnership with trusted community agencies in each population may be more effective.^29,56^ For example, the CDC’s Racial and Ethnic Approaches to Community Health program funds community-based organizations and other institutions to develop community-based, culturally informed strategies that target health disparities in Asian American and other minoritized populations. Since 1999, this program has yielded positive impacts on nutrition access and quality for millions of people.^58^

## Conclusion

In a large, diverse population of low-income Asian Americans, we observed high levels of food insecurity and low levels of participation in CalFresh, California’s implementation of the SNAP program, both of which varied across Asian origin groups during our study period. Our findings suggest that policies supporting language accessibility and providing language assistance resources to community-based organizations may improve SNAP participation among Asian Americans with limited English proficiency. Collecting and reporting disaggregated data by Asian origin group to refine targeting of outreach and interventions may be an important step toward improving nutrition assistance program impact among Asian Americans.

## Supplementary Material

s1

s2

## Figures and Tables

**EXHIBIT 2:**
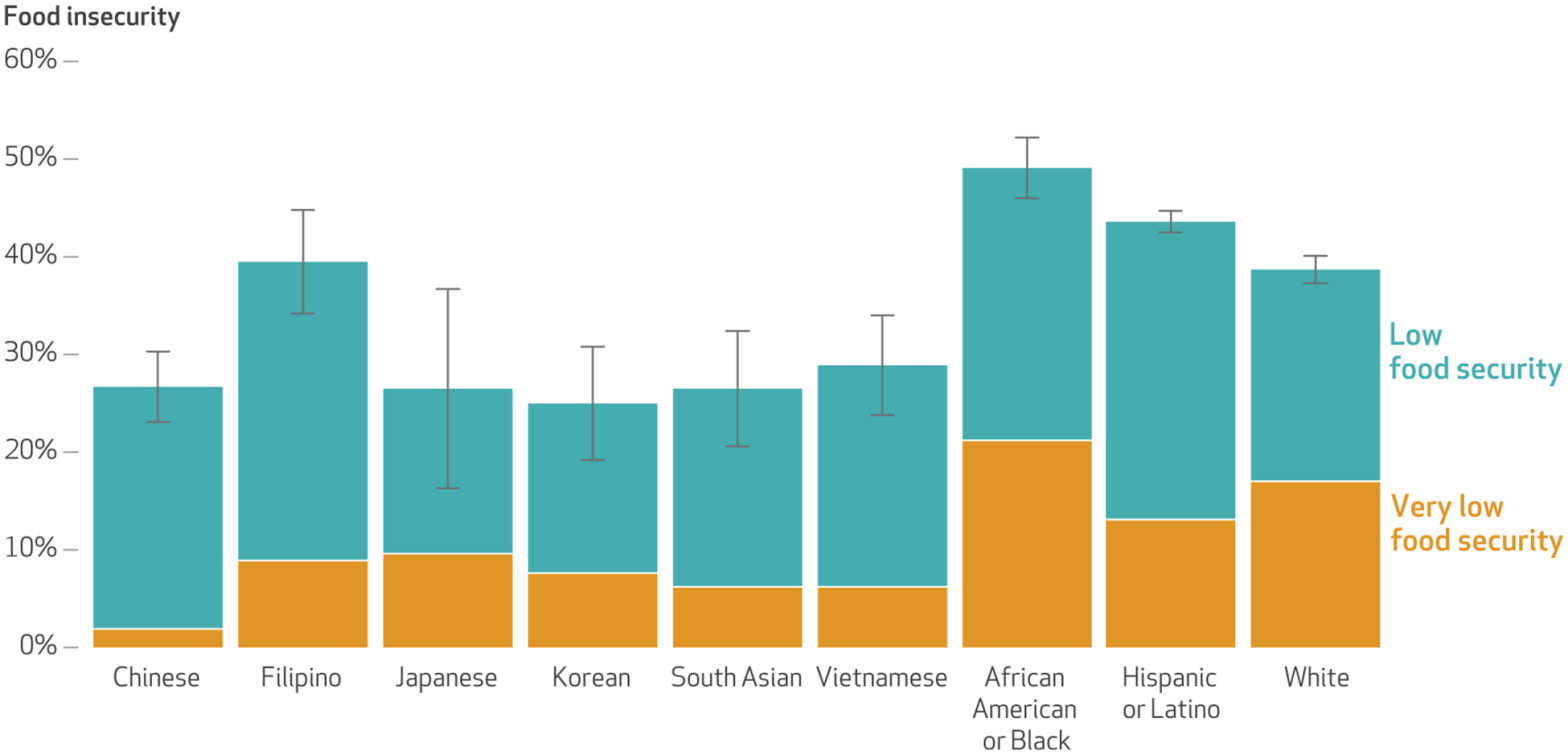
Prevalence and level of food insecurity among low-income California Health Interview Survey respondents, by Asian origin group and other racial and ethnic group, 2011–20 **SOURCE** Authors’ analyses of pooled 2011–20 California Health Interview Survey data. **NOTES** Sample sizes are in the exhibit 1 notes. Data for this exhibit include those with household incomes of less than 200 percent of the federal poverty level. The California Health Interview Survey did not assess food security status among the entire sample; rather, it asked only respondents with household income less than 200 percent of poverty about their food security status. Food insecurity among respondents with household income less than 200 percent of poverty was calculated by dividing the number of respondents who were asked about food security status and indicated food insecurity (low or very low food security) by the number of respondents who were asked about food security status. The error bars indicate 95% confidence intervals. The sum of respondents indicating low food security and respondents indicating very low food security is equal to the total of those indicating food insecurity. All Asian origin groups had significantly lower food insecurity prevalence compared with African American or Black respondents (*p* < 0.05). Except for Filipino respondents, all Asian origin groups had significantly lower food insecurity prevalence compared with Hispanic or Latino and White respondents (*p* < 0.05). The heterogeneity of the two “other” racial and ethnic groups precluded meaningful interpretation; for completeness, data for these two groups were included in exhibit 1 but not exhibits 2–4.

**EXHIBIT 3:**
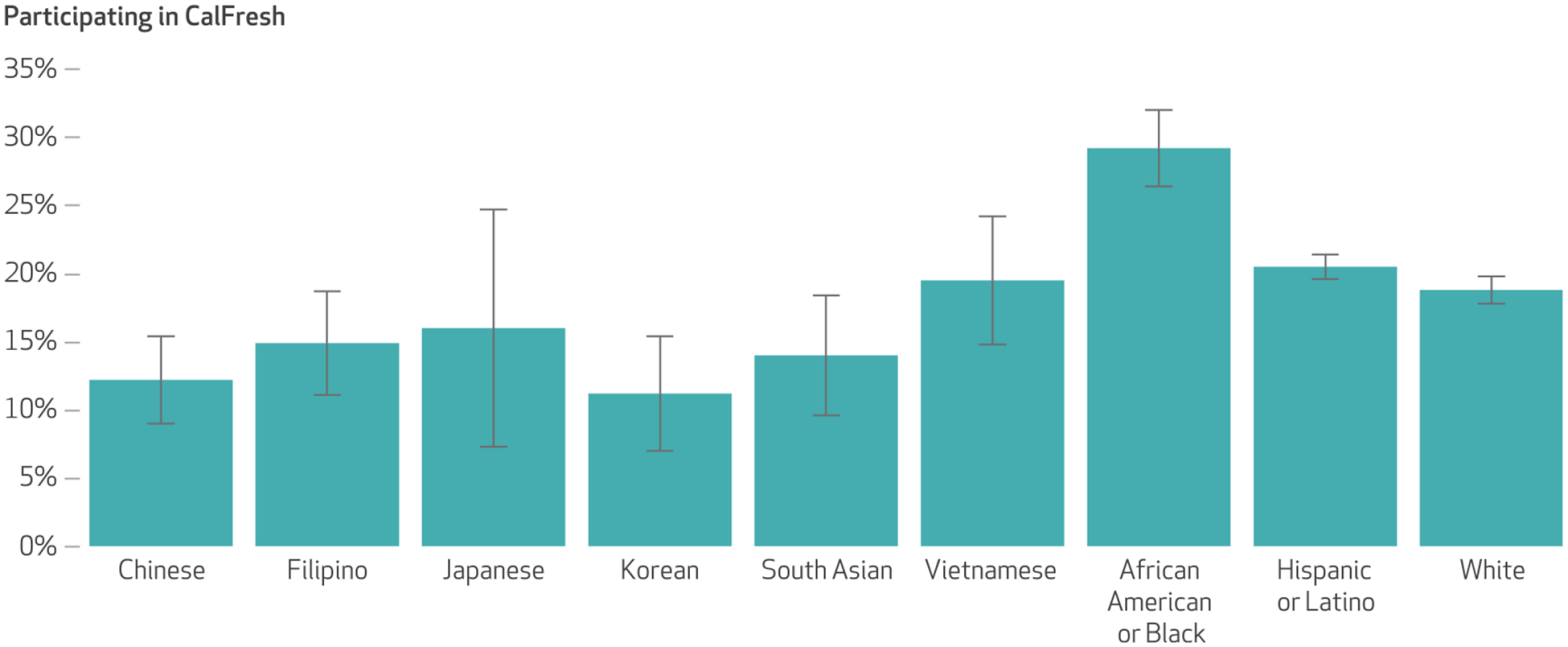
CalFresh participation among low-income California Health Interview Survey respondents, by Asian origin group and other racial and ethnic group, 2011–20 **SOURCE** Authors’ analyses of pooled 2011–20 California Health Interview Survey data. **NOTES** Sample sizes are in the exhibit 1 notes. Data for this exhibit include those with household incomes of 200 percent or less of the federal poverty level. Most CalFresh-receiving households are subject to a gross income determination test, with the maximum gross allowed being 200 percent of poverty. For each group, the prevalence of CalFresh participation was calculated by dividing the number of respondents who had household income at or below 200 percent of poverty, were asked about CalFresh participation, and indicated CalFresh participation by the number of respondents who had household income at or below 200 percent of poverty and were asked about CalFresh participation. The error bars indicate 95% confidence intervals. All Asian origin groups had significantly lower CalFresh participation compared with African American or Black respondents. Chinese, Korean, and South Asian respondents had significantly lower CalFresh participation compared with Hispanic or Latino and White respondents (*p* < 0.05).

**EXHIBIT 1:** Low-Income California Health Interview Survey respondents, by characteristics and CalFresh participation, 2011–20

Characteristics	Total	Not participating in CalFresh	Participating in CalFresh	Unadjusted bivariate odds of CalFresh participation
Race and ethnicity (%)				
Asian origin groups				
Chinese	3.5	3.8	2.1	0.54****
Filipino	2.8	2.9	2.0	0.68**
Japanese	0.4	0.4	0.3	0.74
Korean	1.3	1.4	0.7	0.49***
South Asian	1.0	1.1	0.7	0.63**
Vietnamese	2.5	2.5	2.4	0.94
Other Asian or multiple Asian origin groups^a^	1.3	1.3	1.3	1.00
African American or Black	6.3	5.5	9.1	1.60****
Hispanic or Latino	55.8	55.5	56.9	Ref
White	22.8	23.1	21.3	0.90**
Other^b^	2.4	2.3	3.0	1.29
English proficiency (%)				
Limited English proficiency (speak English not well or not at all)	30.8	30.9	30.1	Ref
English proficient (speak only English, speak English well or very well)	69.2	69.1	69.9	1.04
Age, years (%)				
18–34	36.0	34.8	40.9	Ref
35–49	26.1	24.6	32.2	1.11**
50–64	21.3	22.2	17.8	0.68****
65+	16.6	18.4	9.1	0.42****
Sex (%)				
Male	44.3	46.3	36.7	Ref
Female	55.7	53.7	63.3	1.49****
Education (%)				
Less than a bachelor’s degree	85.4	84.1	90.5	Ref
Bachelor’s degree or higher	14.6	15.9	9.5	0.56****
Employment status (%)				
Unemployed	47.3	44.8	57.2	Ref
Employed (full or part time)	52.7	55.2	42.8	0.61****
Family type (%)				
Without children	87.2	88.4	82.2	Ref
With children	12.8	11.6	17.8	1.65****
Mean family size (no.)	3.76	3.70	4.02	1.08****
Mean household income (% of FPL)	105	111	80	0.34****
Citizenship status (%)				
Noncitizen	28.0	28.0	28.2	Ref
Citizen	72.0	72.0	71.8	0.99

**SOURCE** Authors’ analyses of pooled 2011–20 California Health Interview Survey data. **NOTES** Reference values are 1.00. Unweighted sample sizes were Chinese: 1,936; Filipino: 916; Japanese: 352; Korean: 1,128; South Asian: 409; Vietnamese: 1,730; African American or Black: 3,812; Hispanic or Latino: 24,031; and White: 25,862. Data for this exhibit include those with household incomes of 200 percent or less of the federal poverty level (FPL). All Asian origin and other racial categories are non-Hispanic or Latino. Family size and income are treated as continuous variables in logistic regressions. For race and ethnicity in logistic regressions, Hispanic or Latino respondents were chosen as the reference group, given the small proportion of African American or White respondents identifying as having limited English proficiency.

a Includes Burmese, Cambodian, Hmong, Indonesian, Laotian, Malaysian, Taiwanese, Thai, other Asian Americans not listed here or in the major six origin groups, and those indicating that they identify with 2 or more Asian origin groups.

b Includes American Indian/Alaska Native, Native Hawaiian/Pacific Islander, other race (one race), and other race (multiple races).

** *p* < 0.05

*** *p* < 0.01

**** *p* < 0.001

**EXHIBIT 4:** Adjusted odds ratios of CalFresh participation among low-income Hispanic or Latino and selected Asian American California Health Interview Survey respondents, stratified by English proficiency, 2011–20

	CalFresh participation (odds ratios)	
	Limited English proficiency	English proficient
Hispanic or Latino	Ref	Ref
Asian origin groups		
Chinese	0.98	0.36****
Filipino	1.45	0.87
Korean	1.15	0.36***
South Asian	1.58	0.65
Vietnamese	1.37	0.92

**SOURCE** Authors’ analyses of pooled 2011–20 California Health Interview Survey data. **NOTES** Reference values are 1.00. Respondents with limited English proficiency indicated that they speak English not well or not at all. English-proficient respondents indicated that they speak only English, speak English well, or speak English very well. Models were controlled for age, sex, education, employment status, family type, family size, income, and citizenship status. We did not include separate data for Japanese, African American or Black, and White respondents because of the very low proportions indicating both limited English proficiency and CalFresh participation in each of these groups.

*** *p* < 0.01

**** *p* < 0.001
