# Supplementary material for: Low-Income Asian Americans: High Levels Of Food Insecurity And Low Participation In The CalFresh Nutrition Program
Source: Health Aff (Millwood). Author manuscript; Available in PMC 2024 Jun 18. (PMC11184507; doi:10.1377/hlthaff.2023.00116)
Supplement: s1 [file NIHMS1999877-supplement-s1.pdf]

APPENDIX

Appendix A – Low-Income CHIS respondents, 2011-2020, by Characteristics and CalFresh Participation

Characteristic	Total	Not participating in CalFresh	Participating in CalFresh	Unadjusted bivariate odds of CalFresh participation
**Race and ethnicity (%)**				
Hispanic/Latino	55.8%	55.5%	56.9%	Reference
Chinese	3.5%	3.8%	2.1%	0.54 (0.40 – 0.73)****
Filipino	2.8%	2.9%	2.0%	0.68 (0.49 – 0.93)**
Japanese	0.4%	0.4%	0.3%	0.74 (0.39 – 1.41)
Korean	1.3%	1.4%	0.7%	0.49 (0.31 – 0.75)***
South Asian	1.0%	1.1%	0.7%	0.63 (0.43 – 0.92)**
Vietnamese	2.5%	2.5%	2.4%	0.94 (0.70 – 1.26)
White	22.8%	23.1%	21.3%	0.90 (0.83 – 0.98)**
African American/Black	6.3%	5.5%	9.1%	1.60 (1.37 – 1.87)****
Other Asian/Multiple Asian origin groups	1.3%	1.3%	1.3%	1.00 (0.63 – 1.57)
Other	2.4%	2.3%	3.0%	1.29 (1.08 – 1.55)
**English proficiency (%)**				
Limited English Proficiency (Speak English not well or not at all)	30.8%	30.9%	30.1%	Reference
English Proficient (Speak only English; speak English well or very well)	69.2%	69.1%	69.9%	1.04 (0.96 - 1.13)
**Age (years, %)**				
18-34	36.0%	34.8%	40.9%	Reference
35-49	26.1%	24.6%	32.2%	1.11 (1.01 - 1.23)**
50-64	21.3%	22.2%	17.8%	0.68 (0.62 - 0.76)****
65 and above	16.6%	18.4%	9.1%	0.42 (0.36 - 0.49)****
**Sex (%)**				
Male	44.3%	46.3%	36.7%	Reference
Female	55.7%	53.7%	63.3%	1.49 (1.37 - 1.61)****
**Education (%)**				
Less than a Bachelor’s degree	85.4%	84.1%	90.5%	Reference
Bachelor's degree or higher	14.6%	15.9%	9.5%	0.56 (0.48 - 0.64)****

**Employment status (%)**				
Unemployed	47.3%	44.8%	57.2%	Reference
Employed (full-time or part-time)	52.7%	55.2%	42.8%	0.61 (0.56 - 0.66)****
**Family type (%)**				
Without children	87.2%	88.4%	82.2%	Reference
With children	12.8%	11.6%	17.8%	1.65 (1.48 - 1.83)****
**Mean Family size (number)**	3.76	3.70	4.02	1.08 (1.06 - 1.11)****
**Mean Household Income (percent of FPL)**	105%	111%	80%	0.34 (0.31 - 0.37)****
**Citizenship status (%)**				
Non-citizen	28.0%	28.0%	28.2%	Reference
Citizen	72.0%	72.0%	71.8%	0.99 (0.90 - 1.09)

Source: Authors' analyses of the pooled 2011-2020 California Health Interview Survey data.

Notes: CHIS=California Health Interview Survey. We defined low-income respondents for this analysis as those with household income at or less than 200% of the federal poverty level. For race and ethnicity, except for the Hispanic/Latino category, all remaining categories are non-Hispanic/Latino. Other Asian/Multiple Asian origin groups includes Burmese, Cambodian, Hmong, Indonesian, Laotian, Malaysian, Taiwanese, Thai, other Asians not listed here or in the major six origin groups, and those indicating that they identified with 2+ Asian origin groups. Other includes American Indian/Alaskan Native; Native Hawaiian/Pacific Islander, Other race (one race); Other race (multiple races). Family size and income are treated as continuous variables in logistic regressions. For race and ethnicity in logistic regressions, Hispanic/Latino respondents were chosen as the reference group given the small proportion of White or African American respondents identifying as limited English proficiency.

** $p < 0.05$, *** $p < 0.01$, **** $p < 0.001$.

Appendix B – Low-Income CHIS respondents, 2011-2020, by Asian origin group and other racial and ethnic group

Characteristic	Chinese weighted n=348,188; unweighted n=1,936	Filipino weighted n=275,996; unweighted n=916	Japanese weighted n=40,864; unweighted n=352	Korean weighted n=129,866; unweighted n=1,128	South Asian weighted n=101,244; unweighted n=409	Vietnamese weighted n=247,711; unweighted n=1,730	Hispanic/Latino weighted n=5,587,403; unweighted n=24,031	White weighted n=2,280,566; unweighted n=25,862	African American/Black weighted n=626,973; unweighted n=3,812
**English proficiency (%)**									
Limited English Proficiency (Speak English not well or not at all)	55.5%	9.7%	7.3%	52.9%	6.8%	67.9%	45.9%	1.0%	0.6%
English Proficient (Speak only English; speak English well or very well)	44.5%	90.3%	92.7%	47.1%	93.2%	32.1%	54.1%	99.0%	99.4%
**Age (years, %)**									
18-34	38.0%	35.7%	45.1%	32.1%	58.6%	21.2%	38.4%	30.4%	31.1%
35-49	17.3%	17.4%	12.9%	13.3%	20.0%	24.5%	31.7%	18.0%	22.8%
50-64	18.4%	20.4%	25.5%	18.0%	13.8%	25.4%	19.8%	24.2%	27.0%
65 and above	26.2%	26.5%	16.5%	36.6%	7.7%	29.0%	10.1%	27.4%	19.1%
**Sex (%)**									
Male	42.5%	42.8%	49.2%	37.8%	53.0%	42.4%	45.1%	43.7%	41.1%
Female	57.5%	57.2%	50.8%	62.2%	47.0%	57.6%	54.9%	56.3%	58.9%
**Education (%)**									
Less than a Bachelor's degree	67.3%	61.7%	64.2%	60.6%	54.1%	85.1%	92.8%	77.1%	83.6%
Bachelor's degree or higher	32.7%	38.3%	35.8%	39.4%	45.9%	14.9%	7.2%	22.9%	16.4%
**Employment status (%)**									
Unemployed	54.4%	48.2%	46.0%	59.7%	46.1%	58.8%	40.7%	56.9%	57.2%
Employed (full-time or part-time)	45.6%	51.8%	54.0%	40.3%	53.9%	41.2%	59.3%	43.1%	42.8%
**Family type (%)**									
Without children	91.0%	90.9%	93.0%	97.4%	84.4%	91.0%	83.3%	92.9%	91.2%
With children	9.0%	9.1%	7.0%	2.6%	15.6%	9.0%	16.7%	7.1%	8.8%
**Mean Family size (number)**	3.36	3.91	2.83	2.88	4.00	3.80	4.26	2.90	3.02
**Mean Household Income (percent of FPL)**	103%	108%	1.04 (0.07)	101%	103%	101%	102%	111%	101%

**Citizenship status (%)**									
Non-citizen	34.0%	26.1%	14.4%	33.2%	29.4%	20.3%	42.6%	2.8%	3.4%
Citizen	66.0%	73.9%	85.6%	66.8%	70.6%	79.7%	57.4%	97.2%	96.6%

Source: Authors' analyses of the pooled 2011-2020 California Health Interview Survey data.
Notes: CHIS=California Health Interview Survey. For race and ethnicity, except for the Hispanic/Latino category, all remaining categories are non-Hispanic/Latino.

Appendix C – Unweighted and weighted analytical sample for each CHIS cycle and for the total years of 2011 to 2020

Survey cycle	n (unweighted)	n (weighted)
2011-2012	14,242	2,003,161
2013-2014	12,849	2,114,623
2015-2016	14,547	2,159,019
2017-2018	13,039	2,002,523
2019-2020	9,121	1,812,068
Total (2011-2020)	63,798	10,091,394

Source: Authors' analyses of the pooled 2011-2020 California Health Interview Survey data.
Notes: CHIS=California Health Interview Survey.

Appendix D – Unweighted and weighted analytical sample by race and ethnicity, 2011-2020

Race and ethnicity	n (unweighted)	n (weighted)
Chinese	1,936	348,188
Filipino	916	275,996
Japanese	352	40,864
Korean	1,128	129,866
South Asian	409	101,244
Vietnamese	1,730	247,711
Hispanic/Latino	24,031	5,587,403
White	25,862	2,280,566
African-American/Black	3,812	626,973

Source: Authors' analyses of the pooled 2011-2020 California Health Interview Survey data.
Notes: For race and ethnicity, except for the Hispanic/Latino category, all remaining categories are non-Hispanic/Latino.

Appendix E – CalFresh participation among low-income, food-insecure CHIS respondents by Asian origin group and other racial and ethnic group, 2011-2020

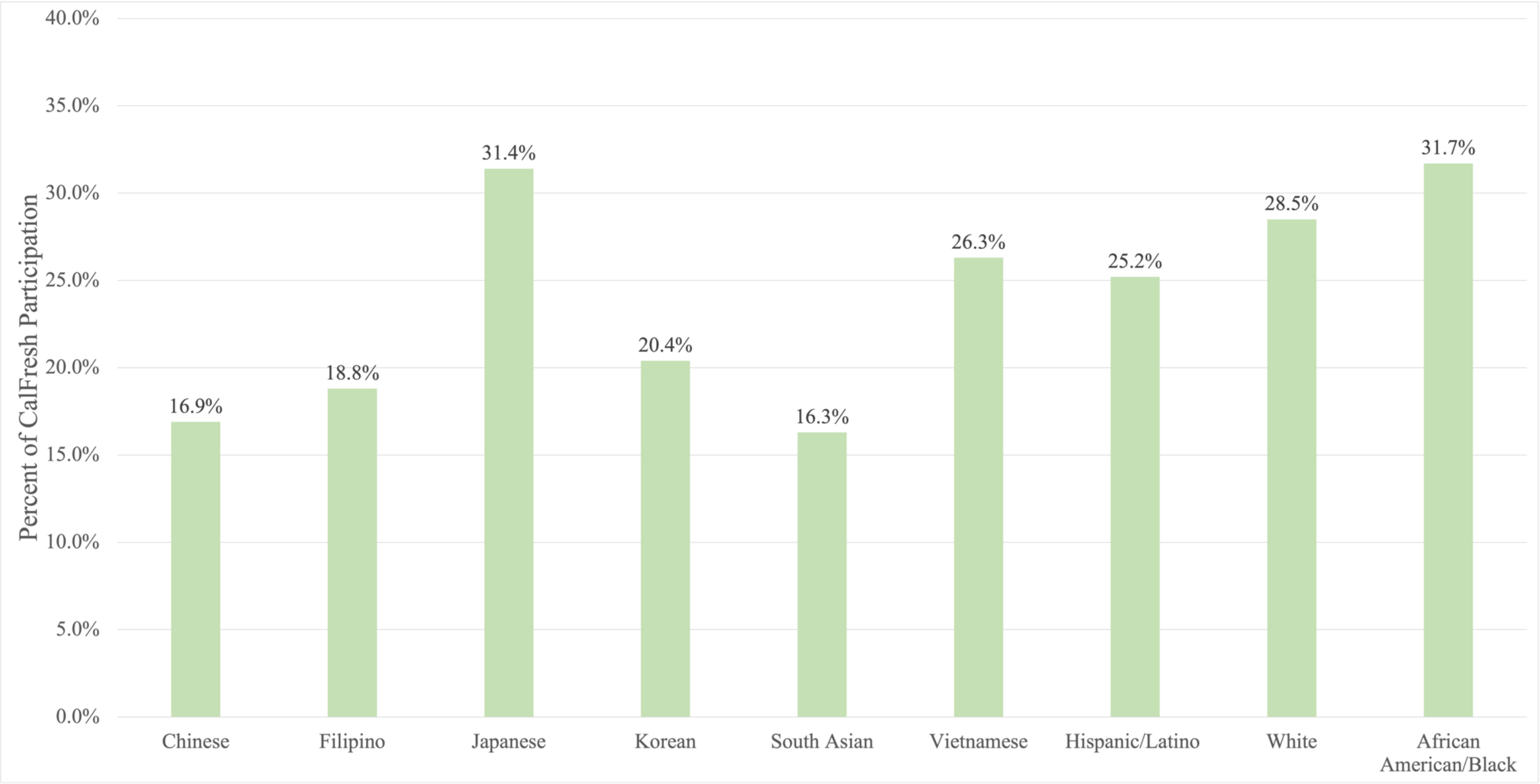

Source: Authors' analyses of the pooled 2011-2020 California Health Interview Survey data.
Notes: CHIS=California Health Interview Survey. For each group, the prevalence of CalFresh participation was calculated by dividing the number of respondents who had household income below 200% of the federal poverty level, were asked about both food insecurity and CalFresh participation, and indicated both food insecurity and CalFresh participation over the number of respondents who had household income below 200% of the federal poverty level, were asked about both food insecurity and CalFresh participation, and indicated food insecurity.

Appendix F – CalFresh participation among low-income Asian American and Hispanic/Latino CHIS respondents with and without limited English proficiency, by race and ethnicity, 2011-2020

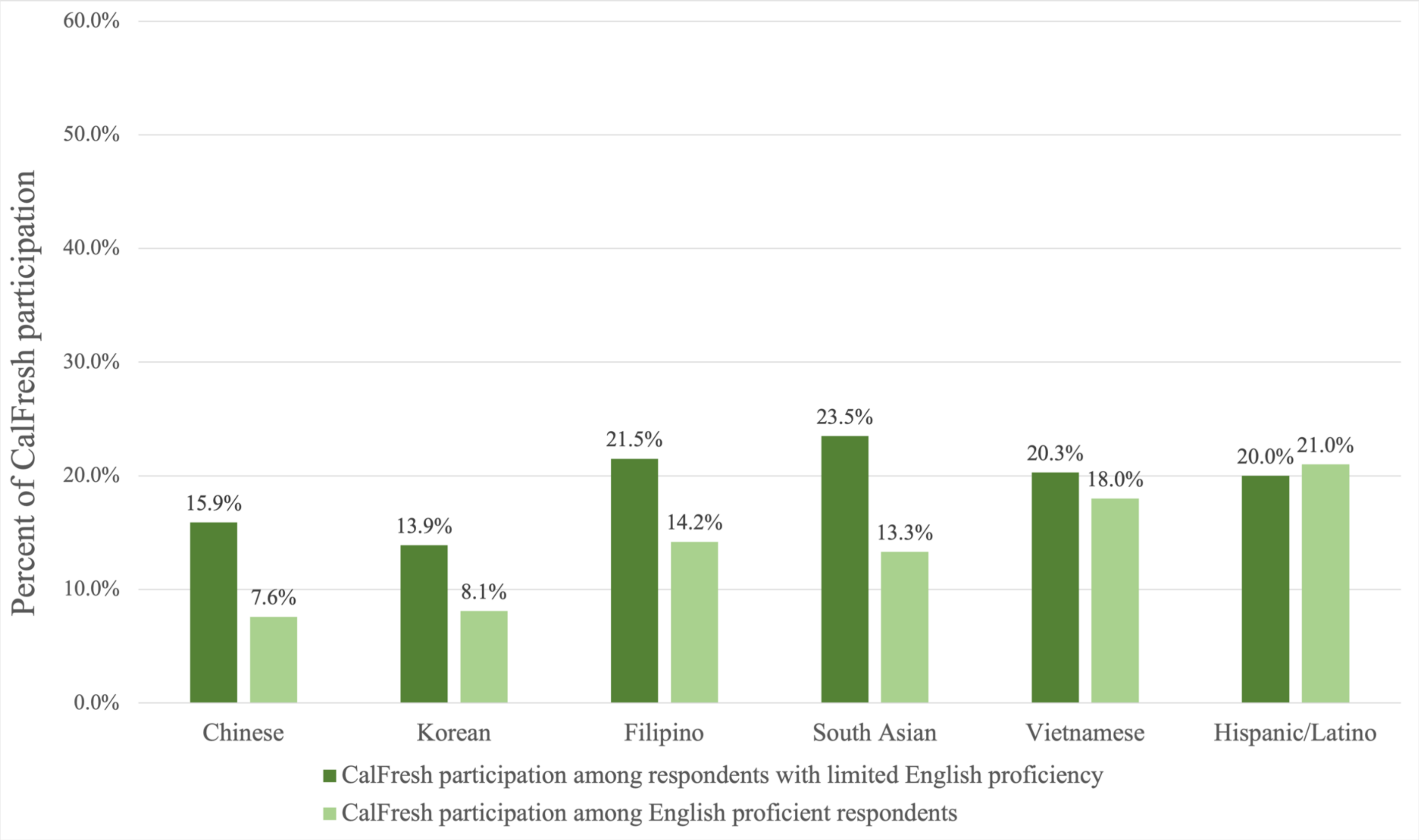

Source: Authors' analyses of the pooled 2011-2020 California Health Interview Survey data.
Notes: CHIS=California Health Interview Survey. We did not include separate data for Japanese, White, and African American/Black respondents due to very low proportion of those indicating both limited English proficiency and CalFresh participation in each of these groups. All comparisons between LEP and English proficient respondents are significant for each racial and ethnic group shown in the exhibit ($p < 0.05$).

Appendix G – Odds ratios of CalFresh participation among low-income CHIS respondents by race and ethnicity, English proficiency, and other characteristics, 2011-2020

Variable	**Model 1 (no interaction term)**	**Model 2 (with race and ethnicity*English proficiency interaction term)**
**Race and ethnicity**		
Hispanic/Latino	Reference	Reference
Chinese	0.65 (0.48 – 0.89)***	0.99 (0.65 – 1.49)
Filipino	0.93 (0.65 – 1.34)	1.49 (0.74 – 3.00)
Korean	0.65 (0.40 – 1.06)	1.15 (0.64 – 2.08)
South Asian	0.68 (0.45 – 1.05)	1.36 (0.03 – 56.24)
Vietnamese	1.08 (0.79 – 1.50)	1.23 (0.83 – 1.81)
Other Asian/Multiple Asian origin groups	0.98 (0.66 – 1.45)	0.80 (0.35 – 1.83)
Other	1.32 (1.18 – 1.48)****	2.38 (0.90 – 6.31)
**English proficiency**		
Limited English Proficiency (Speak English not well or not at all)	Reference	Reference
English Proficient (Speak only English; speak English well or very well)	0.95 (0.84 – 1.07)	1.04 (0.91 – 1.19)
**Race and ethnicity * English proficiency**		
Hispanic/Latino, Not LEP		Reference
Chinese, Not LEP		0.35 (0.20 – 0.62)****
Filipino, Not LEP		0.57 (0.27 – 1.22)
Korean, Not LEP		0.30 (0.13 – 0.67)***
South Asian, Not LEP		0.45 (0.01 – 22.65)
Vietnamese, Not LEP		0.75 (0.40 – 1.41)
Other Asian/Multiple Asian origin groups, Not LEP		1.22 (0.53 – 2.81)
Other, Not LEP		0.53 (0.20 – 1.43)

**Age**		
18-34	Reference	Reference
35-49	1.16 (1.04 – 1.30)***	1.16 (1.04 – 1.30)***
50-64	0.65 (0.58 – 0.73)****	0.65 (0.58 – 0.73)****
65 and above	0.34 (0.29 – 0.41)****	0.33 (0.28 – 0.40)****
**Sex**		
Male	Reference	Reference
Female	1.34 (1.22 – 1.47)****	1.34 (1.23 – 1.47)****
**Education**		
Less than Bachelor's degree	Reference	Reference
Bachelor's degree or above	0.63 (0.54 – 0.73)****	0.64 (0.55 – 0.75)****
**Employment**		
Unemployed	Reference	Reference
Employed	0.54 (0.49 – 0.60)****	0.54 (0.49 – 0.60)****
**Family type**		
Without children	Reference	Reference
With children	1.44 (1.26 – 1.64)****	1.42 (1.25 – 1.62)****
**Family size** (continuous)	1.02 (1.00 – 1.05)	1.03 (1.00 – 1.05)
**Income** (continuous)	0.37 (0.34 – 0.40)****	0.37 (0.34 – 0.40)****
**Citizenship status**		
Non-citizen	Reference	Reference
Citizen	1.18 (1.03 – 1.34)**	1.14 (0.99 – 1.30)

Source: Authors' analyses of the pooled 2011-2020 California Health Interview Survey data.
Notes: CHIS=California Health Interview Survey. LEP=Limited English proficiency. For race and ethnicity, except for the Hispanic/Latino category, all remaining categories are non-Hispanic/Latino. Other Asian/Multiple Asian origin groups includes Japanese (due to insufficient sample of this group that indicates LEP), Burmese, Cambodian, Hmong, Indonesian, Laotian, Malaysian, Taiwanese, Thai, other Asians not listed here or in the major six origin groups, and those indicating that they identified with 2+ Asian origin groups. Other includes American Indian/Alaskan Native; Native Hawaiian/Pacific Islander, Other race (one race); Other race (multiple races). Family size and income are treated as continuous variables in logistic regressions. For race and ethnicity in logistic regressions, Hispanic/Latino respondents were chosen as the reference group given the small proportion of White or African American respondents identifying as LEP. The model with the interaction term explores whether the association between English proficiency and CalFresh participation differs based on race and ethnicity. ** $p < 0.05$, *** $p < 0.01$, **** $p < 0.001$.

Appendix H – Odds ratios of CalFresh participation among low-income Hispanic/Latino and Asian American CHIS respondents, stratified by English proficiency, 2011-2020

Models stratified by English proficiency		
**Racial and ethnic group**	**Limited English proficiency**	**English proficient**
Hispanic/Latino	Reference	Reference
Chinese	0.98 (0.63 – 1.51)	0.36 (0.25 – 0.52)****
Filipino	1.45 (0.73 – 2.87)	0.87 (0.59 – 1.29)
Korean	1.15 (0.65 – 2.03)	0.36 (0.19 – 0.69)***
South Asian	1.58 (0.04 – 63.59)	0.65 (0.41 – 1.04)
Vietnamese	1.37 (0.93 – 2.01)	0.92 (0.54 – 1.56)

Source: Authors' analyses of the pooled 2011-2020 California Health Interview Survey data.
Notes: CHIS=California Health Interview Survey. Models were controlled for age, sex, education, employment status, family type, family size, income, and citizenship status. We did not include separate data for Japanese, White, and African American/Black respondents due to very low proportion of those indicating both limited English proficiency and CalFresh participation in each of these groups.
** $p < 0.05$, *** $p < 0.01$, **** $p < 0.001$.

Appendix I – Odds ratios of CalFresh participation among low-income Hispanic/Latino and Asian American CHIS respondents, stratified by race and ethnicity, 2011-2020

Models stratified by race and ethnicity						
**English proficiency**	**Hispanic/Latino**	**Chinese**	**Filipino**	**Korean**	**South Asian**	**Vietnamese**
Limited English proficiency	Reference	Reference	Reference	Reference	Reference	Reference
English proficient	1.05 (0.92 – 1.22)	0.63 (0.32 – 1.23)	0.61 (0.25 – 1.44)	0.97 (0.37 – 2.53)	0.48 (0.01 – 36.88)	0.74 (0.33 – 1.63)

Source: Authors' analyses of the pooled 2011-2020 California Health Interview Survey data.
Notes: CHIS=California Health Interview Survey. Models were controlled for age, sex, education, employment status, family type, family size, income, and citizenship status. We did not include separate data for Japanese, White, and African American/Black respondents due to very low proportion of those indicating both limited English proficiency and CalFresh participation in each of these groups.
** $p < 0.05$, *** $p < 0.01$, **** $p < 0.001$.

Appendix J – Predicted probabilities of CalFresh participation among low-income CHIS respondents by Asian origin group and English proficiency, 2011-2020

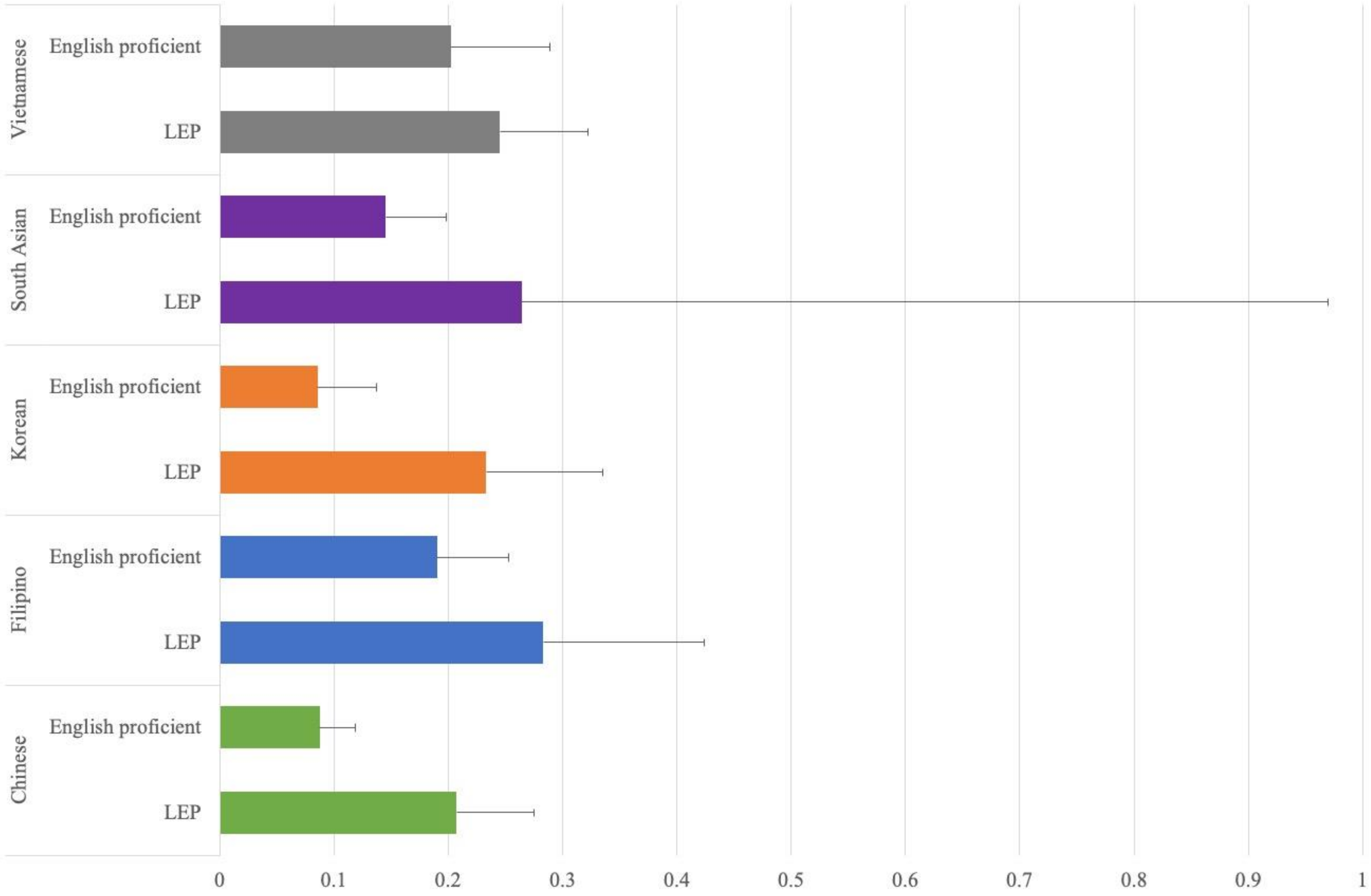

Source: Authors' analyses of the pooled 2011-2020 California Health Interview Survey data.
Notes: CHIS=California Health Interview Survey. LEP=Limited English proficiency. Predicted probability of CalFresh participation by English proficiency and Asian origin group is calculated from weighted multivariable logistic regressions (Appendix G) controlling for age, sex, education, employment status, family type, family size, income, and citizenship status (holding continuous covariates at their means and categorical covariates at their modes). For race and ethnicity, Hispanic/Latino respondents were the reference group.

Appendix K – Low-Income CHIS respondents, 2011-2018, by Characteristics and CalFresh Participation

Characteristics	Total	Not participating in CalFresh	Participating in CalFresh	Unadjusted bivariate odds of CalFresh participation
**Race and ethnicity (%)**				
Hispanic/Latino	55.3%	54.7%	57.9%	Reference
Chinese	3.4%	3.9%	1.7%	0.42 (0.29 – 0.63)****
Filipino	2.7%	3.0%	1.8%	0.58 (0.39 – 0.88)**
Japanese	0.4%	0.4%	0.2%	0.50 (0.16 – 1.51)
Korean	1.3%	1.5%	0.5%	0.33 (0.17 – 0.62)***
South Asian	0.9%	1.0%	0.5%	0.50 (0.29 – 0.86)**
Vietnamese	2.6%	2.6%	2.6%	0.92 (0.67 – 1.26)
White	23.1%	23.6%	21.3%	0.86 (0.77 – 0.95)***
African American/Black	6.3%	5.6%	9.2%	1.55 (1.28 – 1.87)****
Other Asian/Multiple Asian origin groups	1.3%	1.3%	1.2%	0.89 (0.51 – 1.54)
Other	2.5%	2.4%	3.0%	1.20 (0.96 – 1.49)
**English proficiency (%)**				
Limited English Proficiency (Speak English not well or not at all)	33.4%	33.5%	32.9%	Reference
English Proficient (Speak only English; speak English well or very well)	66.6%	66.5%	67.1%	1.03 (0.94 - 1.13)
**Age (years, %)**				
18-34	36.1%	34.6%	42.3%	Reference
35-49	26.9%	25.2%	33.7%	1.09 (0.97 - 1.23)
50-64	21.2%	22.2%	17.3%	0.64 (0.56 - 0.72)****
65 and above	15.8%	18.0%	6.7%	0.31 (0.25 - 0.38)****
**Sex (%)**				
Male	44.6%	46.5%	36.5%	Reference
Female	55.4%	53.5%	63.4%	1.50 (1.37 - 1.65)****
**Education (%)**				
Less than a Bachelor’s degree	85.7%	84.5%	90.8%	Reference
Bachelor’s degree or higher	14.3%	15.5%	9.2%	0.56 (0.46 - 0.67)****
**Employment status (%)**				
Unemployed	46.9%	44.9%	55.4%	Reference
Employed (full-time or part-time)	53.1%	55.1%	44.6%	0.66 (0.59 - 0.73)****

**Family type (%)**				
Without children	92.8%	93.9%	88.5%	Reference
With children	7.17%	6.1%	11.5%	2.00 (1.70 – 2.35)****
**Mean Family size (number)**	3.81	3.72	4.17	1.11 (1.09 - 1.14)****
**Mean Household Income (percent of FPL)**	104%	110%	79%	0.32 (0.29 - 0.35)****
**Citizenship status (%)**				
Non-citizen	29.3%	28.9%	30.8%	Reference
Citizen	70.7%	71.1%	69.2%	0.92 (0.82 - 1.03)

Source: Authors' analyses of the pooled 2011-2018 California Health Interview Survey data.
Notes: CHIS=California Health Interview Survey. We defined low-income respondents for this analysis as those with household income at or less than 200% of the federal poverty level. Our unweighted sample of respondents included: 1,604 Chinese, 721 Filipino, 283 Japanese, 957 Korean, 316 South Asian, and 1,603 Vietnamese respondents. In addition, the unweighted sample included 20,952 Hispanic/Latino, 21,735 non-Hispanic White, and 3,333 non-Hispanic African American/Black respondents. These respondents represented weighted California population estimates of, respectively, 282,459 Chinese, 224,653 Filipinos, 30,897 Japanese, 108,406 Koreans, 75,396 South Asians, 215,844 Vietnamese, 4,538,402 Hispanic/Latino, 1,896,564 White, and 517,108 African American/Black respondents. For race and ethnicity, except for the Hispanic/Latino category, all remaining categories are non-Hispanic/Latino. Other Asian/Multiple Asian origin groups includes Burmese, Cambodian, Hmong, Indonesian, Laotian, Malaysian, Taiwanese, Thai, other Asians not listed here or in the major six origin groups, and those indicating that they identified with 2+ Asian origin groups. Other includes American Indian/Alaskan Native; Native Hawaiian/Pacific Islander, Other race (one race); Other race (multiple races). Family size and income are treated as continuous variables in logistic regressions. For race and ethnicity in logistic regressions, Hispanic/Latino respondents were chosen as the reference group given the small proportion of White or African American respondents identifying as limited English proficiency.
Compared to the analysis with 2011-2020 data, results did not change substantially. Results that were statistically significant in the 2011-2020 data analysis but no longer statistically significant in 2011-2018 data analysis are highlighted. We did not detect any results that were not statistically significant in the 2011-2020 data analysis but then became statistically significant in the 2011-2018 data analysis.
** $p < 0.05$, *** $p < 0.01$, **** $p < 0.001$.

Appendix L – Low-Income CHIS respondents, 2011-2018, by Asian origin group and other racial and ethnic group

	Chinese weighted n=282,459; unweighted n=1,604	Filipino weighted n=224,653; unweighted n=721	Japanese weighted n=30,897; unweighted n=283	Korean weighted n=108,406; unweighted n=957	South Asian weighted n=75,396; unweighted n=316	Vietnamese weighted n=215,844; unweighted n=1,603	Hispanic/Latino weighted n=4,538,402; unweighted n=20,952	White weighted n=1,896,564; unweighted n=21,735	African American/Black weighted n=517,108; unweighted n=3,333
**English proficiency (%)**									
Limited English Proficiency (Speak English not well or not at all)	57.3%	10.7%	8.7%	55.0%	8.1%	71.9%	50.6%	0.8%	0.4%
English Proficient (Speak only English; speak English well or very well)	42.7%	89.3%	91.3%	45.0%	91.9%	28.1%	49.4%	99.2%	99.6%
**Age (years, %)**									
18-34	38.0%	37.2%	41.0%	32.2%	57.8%	19.3%	38.1%	31.4%	32.4%
35-49	17.6%	15.8%	15.1%	12.8%	20.2%	25.1%	32.7%	18.8%	23.4%
50-64	18.0%	21.4%	24.7%	17.6%	14.8%	26.2%	19.6%	24.2%	26.7%
65 and above	26.3%	25.6%	19.1%	37.4%	7.3%	29.3%	9.6%	25.7%	17.5%
**Sex (%)**									
Male	42.3%	43.3%	52.4%	37.6%	53.7%	42.3%	45.6%	43.8%	41.0%
Female	57.7%	56.7%	47.6%	62.4%	46.3%	57.7%	54.4%	56.2%	59.0%
**Education (%)**									
Less than a Bachelor's degree	67.4%	60.9%	63.6%	61.2%	52.5%	85.4%	93.3%	77.2%	84.0%
Bachelor's degree or higher	32.6%	39.1%	36.4%	38.8%	47.5%	14.6%	6.7%	22.8%	16.0%
**Employment status (%)**									
Unemployed	53.1%	48.6%	46.9%	59.9%	46.7%	58.6%	40.2%	56.3%	57.2%
Employed (full-time or part-time)	46.9%	51.4%	53.1%	40.1%	53.3%	41.4%	59.8%	43.7%	42.8%
**Family type (%)**									
Without children	95.1%	95.3%	97.8%	99.6%	91.5%	96.0%	90.7%	95.2%	95.8%
With children	4.9%	4.7%	2.2%	0.4%	8.5%	4.0%	9.3%	4.8%	4.2%
**Mean Family size (number)**	3.38	3.97	2.84	2.91	4.12	3.82	4.31	2.95	3.05
**Mean Household Income (percent of FPL)**	103%	109%	107%	99%	108%	100%	102%	111%	100%
**Citizenship status (%)**									
Non-citizen	34.1%	27.2%	14.8%	33.4%	25.0%	19.7%	45.3%	2.9%	3.4%
Citizen	65.9%	72.8%	85.2%	66.6%	75.0%	80.3%	54.7%	97.1%	96.6%

Source: Authors' analyses of the pooled 2011-2018 California Health Interview Survey data.

Notes: CHIS=California Health Interview Survey. For race and ethnicity, except for the Hispanic/Latino category, all remaining categories are non-Hispanic/Latino. Compared to the analysis with 2011-2020 data, results did not change substantially.

Appendix M – Prevalence and levels of food insecurity among low-income CHIS respondents, by Asian origin group and other racial and ethnic group, 2011-2018

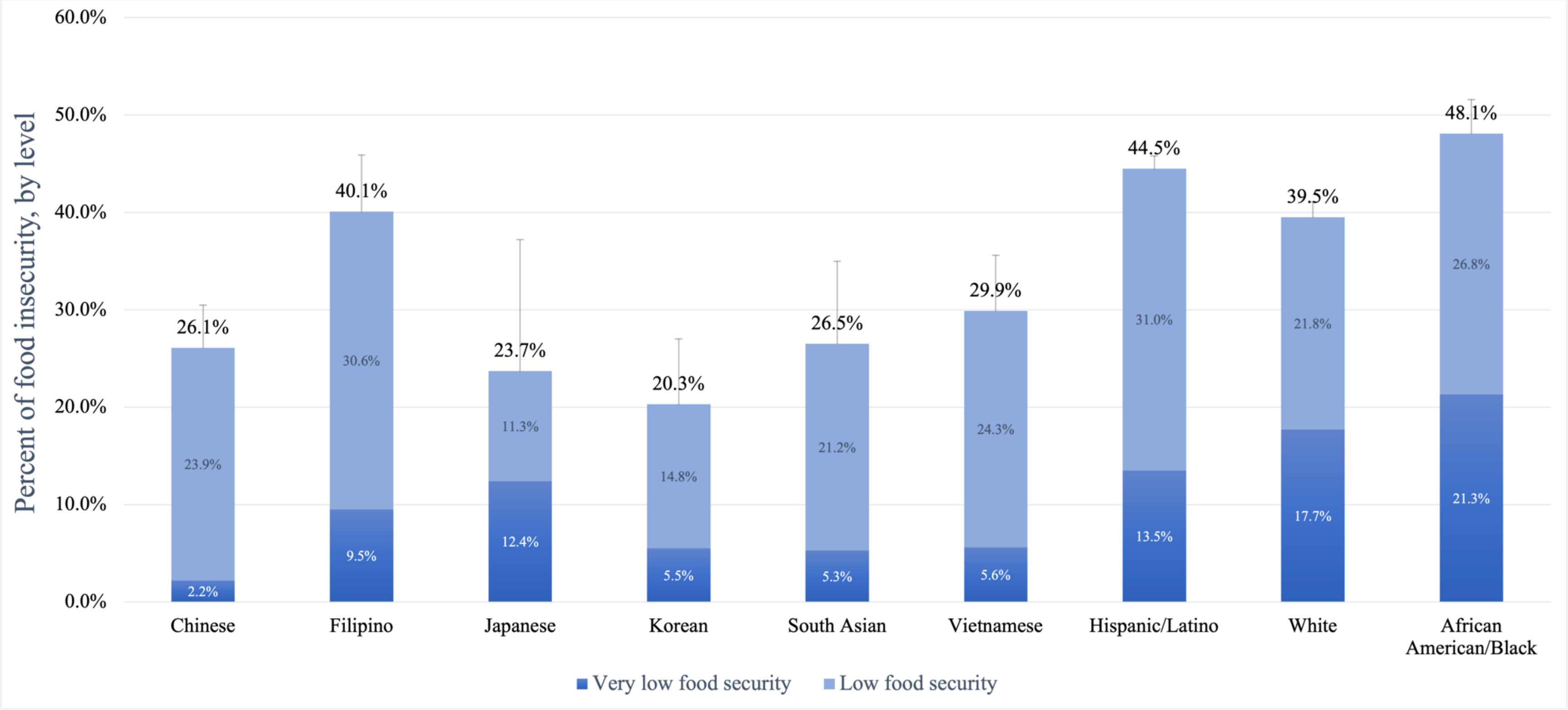

Source: Authors' analyses of the pooled 2011-2018 California Health Interview Survey data.

Notes: CHIS=California Health Interview Survey. CHIS did not assess food security status among the entire sample; rather, they asked only respondents with household income less than 200% of the federal poverty level about their food security status. For each group, food insecurity among respondents with household income less than 200% of the federal poverty level was calculated by dividing the number of respondents who were asked about food security status and indicated food insecurity (low or very low) over the number of respondents who were asked about food security status. The error bars indicate 95% confidence intervals. The sum of those indicating very low food security and those indicating low food security is equal to the total of those indicating food insecurity. Compared to the analysis with 2011-2020 data, results did not change substantially. Similar to 2011-2020 data, Filipino adults remain the Asian origin group with highest total prevalence of food insecurity; Japanese adults remain the Asian origin group with highest severity of food insecurity.

Except for Filipino respondents, all Asian origin groups have significantly lower food insecurity prevalence compared to Hispanic/Latino and White respondents ($p < 0.05$). All Asian origin groups have significantly lower food insecurity prevalence compared to African American/Black respondents ($p < 0.05$).

Appendix N – CalFresh participation among low-income CHIS respondents, by Asian origin group and other racial and ethnic group, 2011-2018

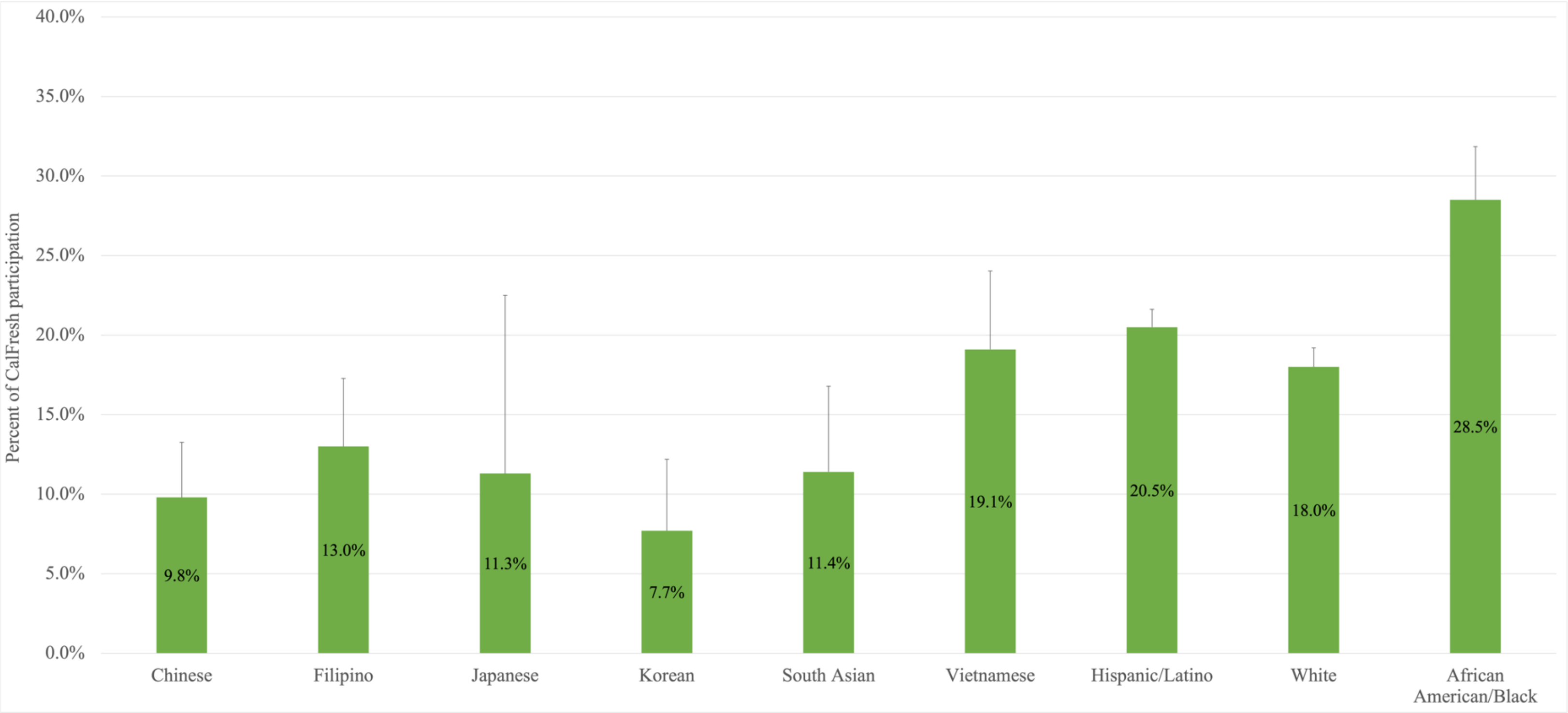

Source: Authors' analyses of the pooled 2011-2020 California Health Interview Survey data.
Notes: CHIS=California Health Interview Survey. For the California-implemented version of the CalFresh (CalFresh), most CalFresh-receiving households are subject to a gross income determination test, with the maximum gross allowed being 200% of the federal poverty level. For each group, the prevalence of CalFresh participation was calculated by dividing the number of respondents who had household income at or below 200% of the federal poverty level, were asked about CalFresh participation, and indicated CalFresh participation over the number of respondents who had household income at or below 200% of the federal poverty level and were asked about CalFresh participation. Compared to the analysis with 2011-2020 data, results did not change substantially. We observed slightly lower CalFresh participation among Asian respondents in the 2011-2018 data when compared to 2011-2020 data. Similar to 2011-2020 data, Korean and Chinese adults remain the Asian origin groups with lowest CalFresh participation. Vietnamese adults remain the Asian origin group with highest participation. The error bars indicate 95% confidence intervals. All Asian origin groups have significantly lower CalFresh participation compared to African American/Black respondents (28.5%). Chinese, Filipino, Korean, and South Asian respondents have significantly lower CalFresh participation compared to Hispanic/Latino (20.5%) and White (18.0%) respondents ($p < 0.05$).

Appendix O – CalFresh participation among low-income, food-insecure CHIS respondents by Asian origin group and other racial and ethnic group, 2011-2018

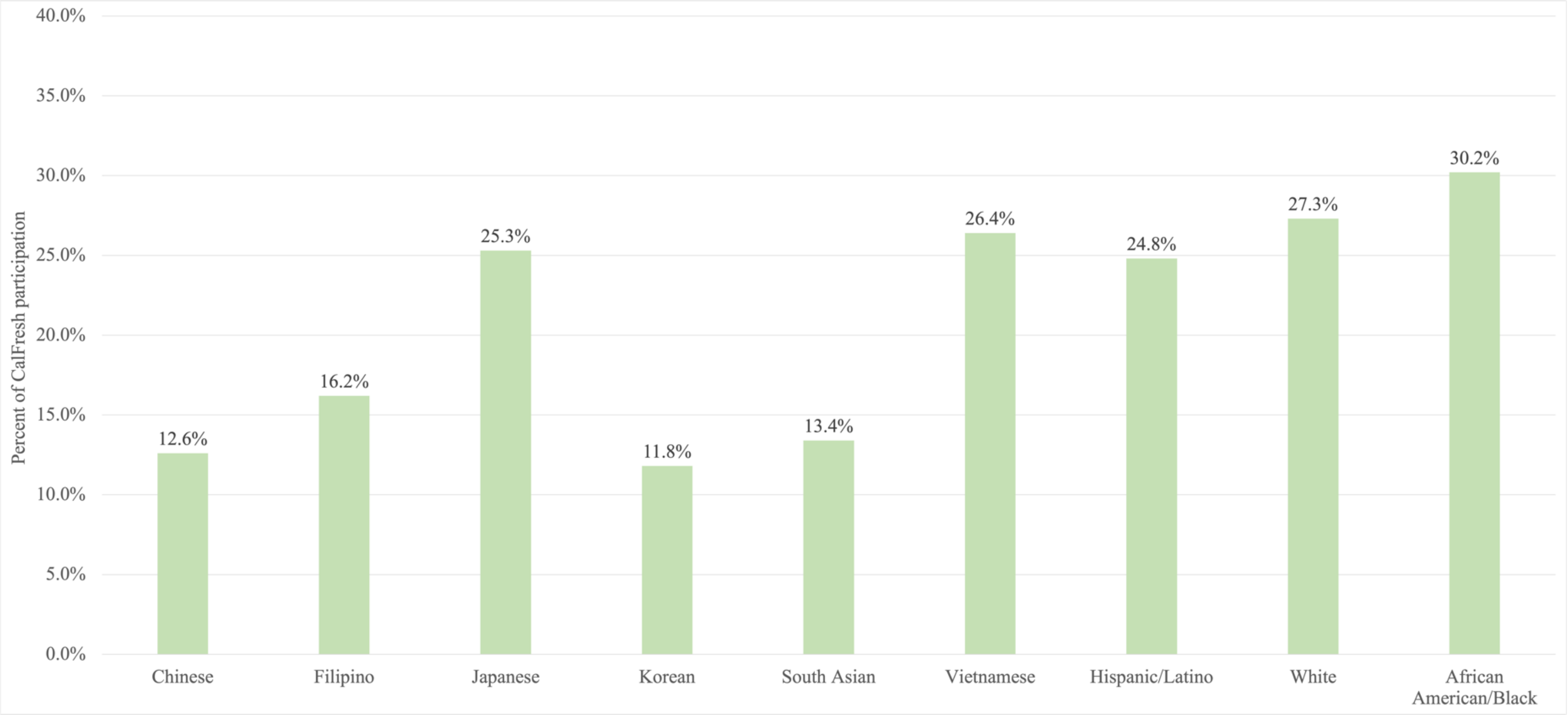

Source: Authors' analyses of the pooled 2011-2018 California Health Interview Survey data.
Notes: CHIS=California Health Interview Survey. For each group, the prevalence of CalFresh participation was calculated by dividing the number of respondents who had household income below 200% of the federal poverty level, were asked about both food insecurity and CalFresh participation, and indicated both food insecurity and CalFresh participation over the number of respondents who had household income below 200% of the federal poverty level, were asked about both food insecurity and CalFresh participation, and indicated food insecurity. In the analysis of data from 2011-2020, CalFresh participation among food insecure respondents varied across Asian origin groups and was ranked in ascending order as follows: South Asian, Chinese, Filipino, Korean, Vietnamese, and Japanese. When analyzing data from 2011-2018, the order of ranking slightly differed and was as follows: Korean, Chinese, South Asian, Filipino, Japanese, and Vietnamese.

Appendix P – CalFresh participation among low-income Asian American and Hispanic/Latino CHIS respondents with and without limited English proficiency, by race and ethnicity, 2011-2018

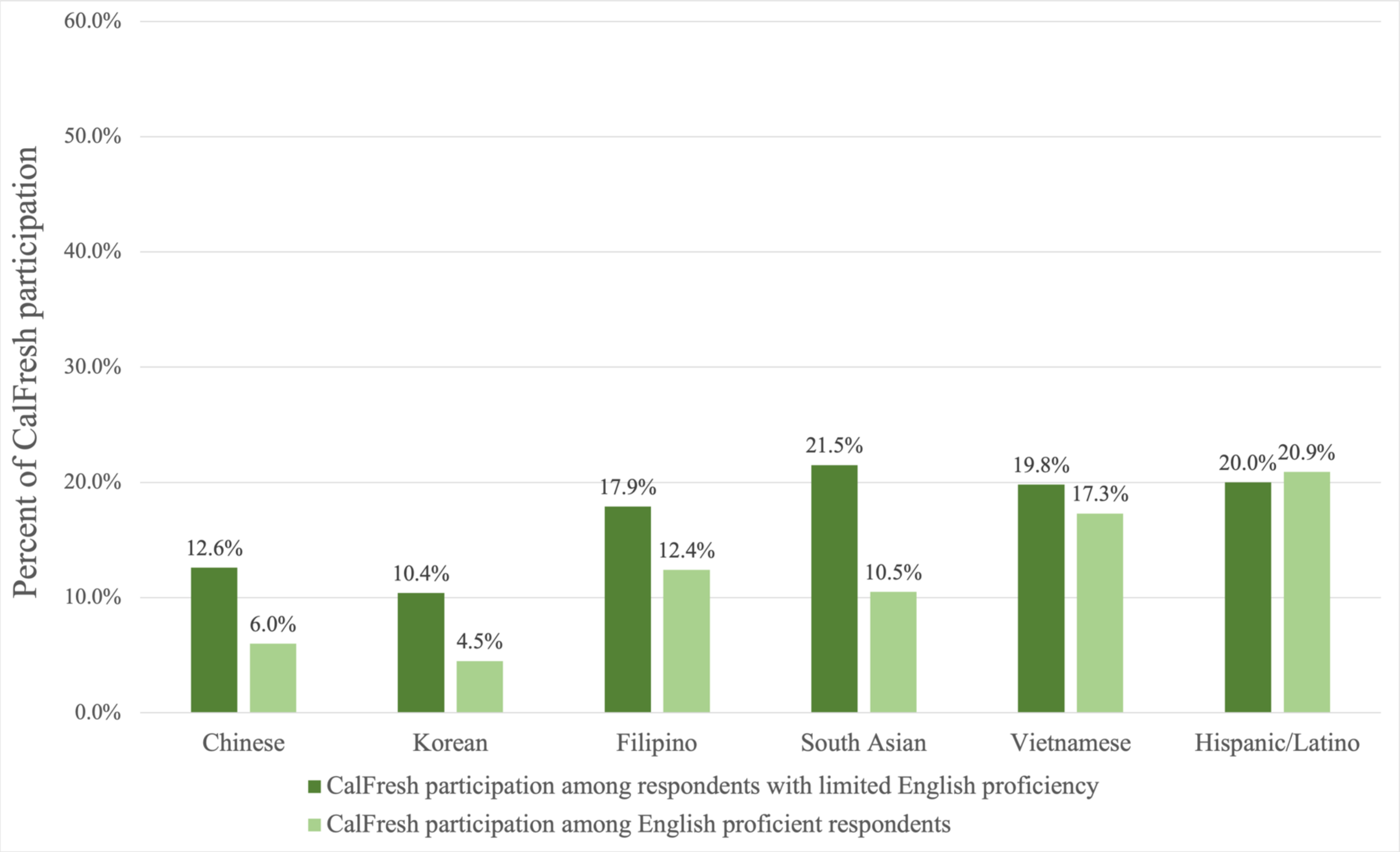

Source: Authors' analyses of the pooled 2011-2020 California Health Interview Survey data.
Notes: CHIS=California Health Interview Survey. We did not include separate data for Japanese, White, and African American/Black respondents due to very low proportion of those indicating both limited English proficiency (LEP) and CalFresh participation in each of these groups. Compared to the analysis with 2011-2020 data, results did not change substantially. Similar to what we observed in the 2011-2020 data, respondents with LEP had higher CalFresh participation compared to English proficient respondents. The magnitudes of differences varied considerably across Asian origin groups. For example, LEP Chinese respondents participated at more than twice the rate of English proficient Chinese respondents (12.6% vs 6.0%, respectively), while the difference among LEP and English proficient Vietnamese respondents was much smaller (19.8% and 17.3%, respectively). This pattern of higher CalFresh participation among LEP respondents, however, did not hold for Hispanic/Latino respondents. All comparisons between LEP and English proficient respondents are significant for each racial and ethnic group shown in the exhibit ($p < 0.05$).

Appendix Q - Odds ratios of CalFresh participation among low-income Hispanic/Latino and Asian American CHIS respondents, stratified by English proficiency, 2011-2018

Models stratified by English proficiency		
**Racial and ethnic group**	**Limited English proficiency**	**English proficient**
Hispanic/Latino	Reference	Reference
Chinese	0.81 (0.46 – 1.44)	0.28 (0.16 – 0.47)****
Filipino	1.25 (0.53 – 2.93)	0.78 (0.49 – 1.24)
Korean	0.98 (0.46 – 2.08)	0.18 (0.05 – 0.60)***
South Asian	1.36 (0.00 – 1951.52)	0.51 (0.25 – 1.04)
Vietnamese	1.47 (0.96 – 2.26)	0.87 (0.44 – 1.73)

Source: Authors' analyses of the pooled 2011-2018 California Health Interview Survey data.
Notes: CHIS=California Health Interview Survey. Models were controlled for age, sex, education, employment status, family type, family size, income, and citizenship status. We did not include separate data for Japanese, White, and African American/Black respondents due to very low proportion of those indicating both limited English proficiency and CalFresh participation in each of these groups.
Compared to the analysis with 2011-2020 data, results did not change substantially. Results that were statistically significant in the 2011-2020 data analysis stayed significant in the 2011-2018 data analysis. We did not detect any results that were not statistically significant in the 2011-2020 data analysis but then became statistically significant in the 2011-2018 data analysis.
** $p < 0.05$, *** $p < 0.01$, **** $p < 0.001$.

Appendix R - Odds ratios of CalFresh participation among low-income Hispanic/Latino and Asian American CHIS respondents, stratified by race and ethnicity, 2011-2018

Models stratified by race and ethnicity						
**English proficiency**	**Hispanic/Latino**	**Chinese**	**Filipino**	**Korean**	**South Asian**	**Vietnamese**
Limited English proficiency	Reference	Reference	Reference	Reference	Reference	Reference
English proficient	1.13 (0.96 – 1.34)	0.40 (0.16 – 1.04)	0.59 (0.22 – 1.54)	0.97 (0.37 – 2.53)	0.80 (0.00 – 1856.22)	0.64 (0.23 – 1.81)

Source: Authors' analyses of the pooled 2011-2018 California Health Interview Survey data.
Notes: CHIS=California Health Interview Survey. Models were controlled for age, sex, education, employment status, family type, family size, income, and citizenship status. We did not include separate data for Japanese, White, and African American/Black respondents due to very low proportion of those indicating both limited English proficiency and CalFresh participation in each of these groups.
Compared to the analysis with 2011-2020 data, results did not change substantially. Results that were statistically significant in the 2011-2020 data analysis stayed significant in the 2011-2018 data analysis. We did not detect any results that were not statistically significant in the 2011-2020 data analysis but then became statistically significant in the 2011-2018 data analysis.

Appendix S – Predicted probabilities of CalFresh participation among low-income CHIS respondents by Asian origin group and English proficiency, 2011-2018

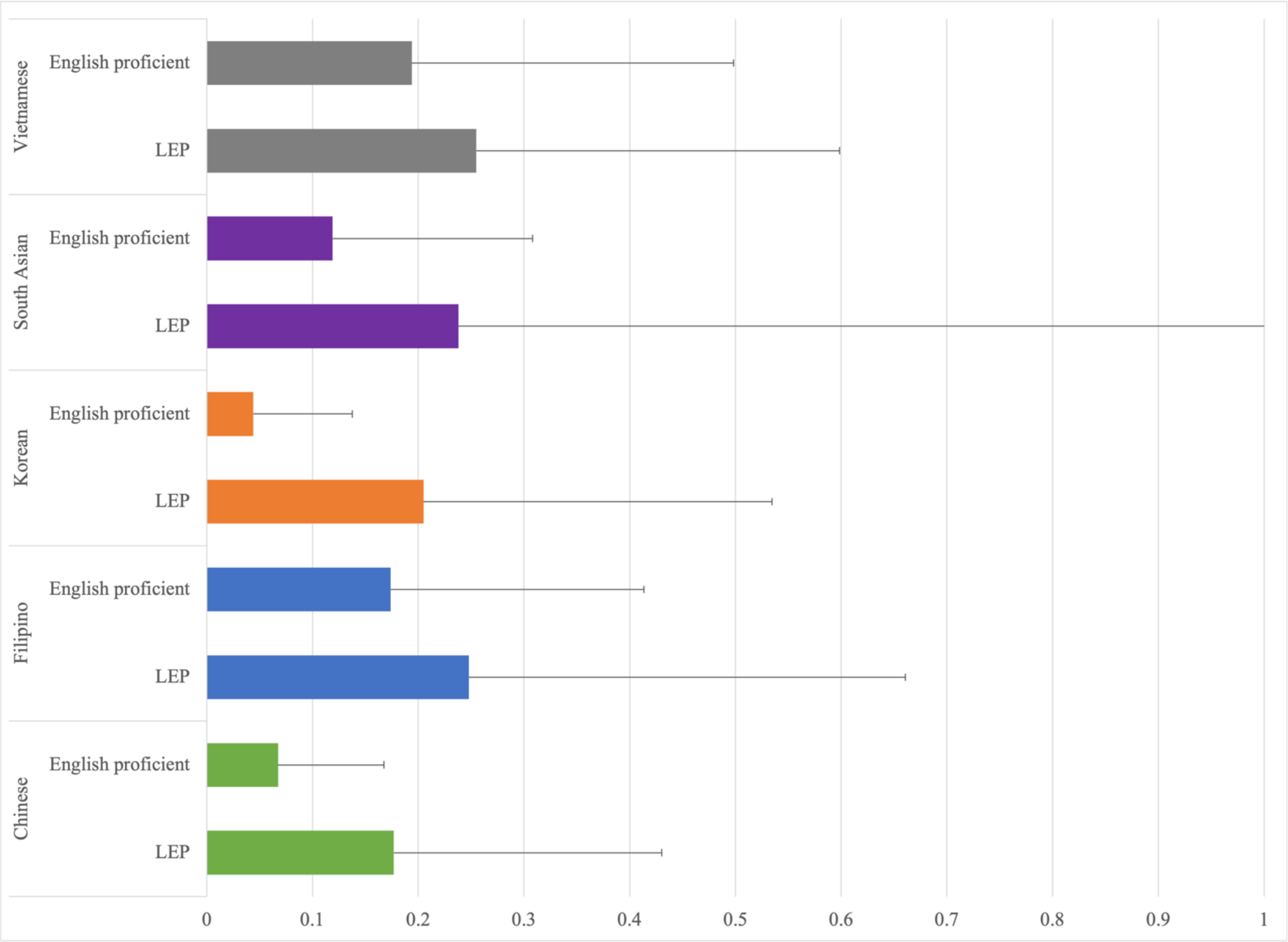

Source: Authors' analyses of the pooled 2011-2018 California Health Interview Survey data.
Notes: CHIS=California Health Interview Survey. LEP=Limited English proficiency. Predicted probability of CalFresh participation by English proficiency and Asian origin group is calculated from weighted multivariable logistic regressions (Appendix T Model 2) controlling for age, sex, education, employment status, family type, family size, income, and citizenship status (holding continuous covariates at their means and categorical covariates at their modes). For race and ethnicity, Hispanic/Latino respondents were the reference group. Compared to the analysis with 2011-2020 data, results did not change substantially. Similar to what we observed in the 2011-2020 data, among English proficient respondents, Korean respondents had the lowest probability and Vietnamese respondents had the highest probability of CalFresh participation. However, among LEP respondents, Chinese respondents had the lowest probability and Filipino had the highest probability of CalFresh participation.

Appendix T - Odds ratios of CalFresh participation among low-income CHIS respondents by race and ethnicity, English proficiency, and other sociodemographic characteristics, 2011-2018

Variable	**Model 1 (no interaction term)**	**Model 2 (with race and ethnicity*English proficiency interaction term)**
**Race and ethnicity**		
Hispanic/Latino	Reference	Reference
Chinese	0.53 (0.36 – 0.80)***	0.84 (0.49 – 1.43)
Filipino	0.83 (0.53 – 1.28)	1.28 (0.53 – 3.13)
Korean	0.46 (0.23 – 0.90)**	1.01 (0.46 – 2.18)
South Asian	0.55 (0.30 – 1.03)	1.22 (0.00 – 1734.69)
Vietnamese	1.14 (0.79 – 1.64)	1.33 (0.86 – 2.06)
Other Asian	0.84 (0.50 – 1.39)	0.60 (0.22 – 1.61)
Other	1.29 (1.12 – 1.48)****	0.80 (0.25 – 2.58)
**English proficiency**		
Limited English Proficiency (Speak English not well or not at all)	Reference	Reference
English Proficient (Speak only English; speak English well or very well)	1.01 (0.87 – 1.17)	1.09 (0.93 – 1.28)
**Race and ethnicity * English proficiency**		
Hispanic/Latino, Not LEP		Reference
Chinese, Not LEP		0.31 (0.14 – 0.69)***
Filipino, Not LEP		0.59 (0.23 – 1.53)
Korean, Not LEP		0.16 (0.04 – 0.63)***
South Asian, Not LEP		0.40 (0.00 – 769.29)
Vietnamese, Not LEP		0.65 (0.29 – 1.46)
Other Asian/Multiple Asian origin groups Not LEP		1.41 (0.50 – 3.95)
Other, Not LEP		1.57 (0.48 – 5.14)

**Age**		
18-34	Reference	Reference
35-49	1.12 (0.99 – 1.28)	1.12 (0.98 – 1.28)
50-64	0.62 (0.54 – 0.72)****	0.62 (0.54 – 0.72)****
65 and above	0.27 (0.21 – 0.34)****	0.26 (0.20 – 0.33)****
**Sex**		
Male	Reference	Reference
Female	1.37 (1.23 – 1.53)****	1.37 (1.23 – 1.52)****
**Education**		
Less than Bachelor's degree	Reference	Reference
Bachelor's degree or above	0.65 (0.54 – 0.79)****	0.67 (0.55 – 0.81)****
**Employment**		
Unemployed	Reference	Reference
Employed	0.57 (0.51 – 0.65)****	0.57 (0.50 – 0.65)****
**Family type**		
Without children	Reference	Reference
With children	1.67 (1.39 – 2.01)****	1.67 (1.39 – 2.01)****
**Family size** (continuous)	1.05 (1.02 – 1.08)***	1.05 (1.02 – 1.08)***
**Income** (continuous)	0.35 (0.31 – 0.39)****	0.35 (0.31 – 0.39)****
**Citizenship status**		
Non-citizen	Reference	Reference
Citizen	1.12 (0.96 – 1.32)	1.09 (0.93 – 1.28)

Source: Authors' analyses of the pooled 2011-2020 California Health Interview Survey data.
Notes: CHIS=California Health Interview Survey. LEP=Limited English proficiency. For race and ethnicity, except for the Hispanic/Latino category, all remaining categories are non-Hispanic/Latino. Other Asian/Multiple Asian origin groups includes Japanese (due to insufficient sample of this group that indicates LEP), Burmese, Cambodian, Hmong, Indonesian, Laotian, Malaysian, Taiwanese, Thai, other Asians not listed here or in the major six origin groups, and those indicating that they identified with 2+ Asian origin groups. Other includes American Indian/Alaskan Native; Native Hawaiian/Pacific Islander, Other race (one race); Other race (multiple races). Family size and income are treated as continuous variables in logistic regressions. For race and ethnicity in logistic regressions, Hispanic/Latino respondents were chosen as the reference group given the small proportion of White or African American respondents identifying as LEP. The model with the interaction term explores whether the association between English proficiency and CalFresh participation differs based on race and ethnicity.

Compared to the analysis with 2011-2020 data, results did not change substantially. Results that were statistically significant in the 2011-2020 data analysis but no longer statistically significant in 2011-2018 data analysis are highlighted. Results that were not statistically significant in the 2011-2020 data analysis but then became statistically significant in the 2011-2018 data analysis are in red.

** $p < 0.05$, *** $p < 0.01$, **** $p < 0.001$.
